# The Implementation Process for Pharmacogenomic Testing for Cancer-Targeted Therapies

**DOI:** 10.3390/jpm8040032

**Published:** 2018-10-01

**Authors:** Ann Chen Wu, Kathleen M. Mazor, Rachel Ceccarelli, Stephanie Loomer, Christine Y. Lu

**Affiliations:** 1 PRecisiOn Medicine Translational Research (PROMoTeR) Center, Department of Population Medicine, Harvard Pilgrim Health Care Institute and Harvard Medical School, 401 Park Drive, Suite 401, Boston, MA 02215, USA; rceccare@bu.edu (R.C.); stephaniejt28@gmail.com (S.L.); christine_lu@harvardpilgrim.org (C.Y.L.); 2 Meyers Primary Care Institute, 385 Grove Street, Worcester, MA 01605, USA; Kathy.Mazor@meyersprimary.org

**Keywords:** pharmacogenomics, implementation, cancer

## Abstract

Recent advances in genomic medicine have led to the availability of genomic tests that have the potential to improve population health, yet the process for obtaining these tests and getting them reimbursed by insurers has not been described. The objective of this study was to describe the process of ordering pharmacogenomic tests by interviewing providers, patients, and laboratories about cancer-related pharmacogenomic tests. We interviewed patients who were prescribed, providers who prescribed medications that should be guided by pharmacogenomic testing, and individuals from diagnostic laboratories. A total of 10 providers, 16 patients, and eight diagnostic laboratories described logistical and insurance issues relating to ordering and receiving pharmacogenomic tests and medications. We found that the process of ordering pharmacogenomic tests is time-consuming, expensive, and complex. Ordering pharmacogenomic tests is quite different across institutions. Even in the same institution, multiple providers can order the test. Once the provider places the order for the pharmacogenomic test, the laboratory receives the request and usually begins testing without knowing how the test will be paid for. Next, the laboratory completes the pharmacogenomic testing and the results of the tests are reported to providers, patients, or placed directly in the medical record. In conclusion, processes related to ordering and obtaining insurance coverage for pharmacogenomic tests varies greatly across institutions and is time-consuming.

## 1. Introduction

Genomic medicine is exploding with discoveries that could improve cancer care, and many pharmacogenomic tests are becoming available for use in clinical practice [1,2]. Nevertheless, translation of new genomic technologies into clinical practice has been slower than hoped [3,4]. As of January 2018, 54,334 tests for 10,999 conditions and 16,419 genes were available [5]. The U.S. Food and Drug Administration (FDA) has included pharmacogenomic information in the drug labels of more than 100 drugs, with 31% being oncology therapy [6]. For example, clinical guidelines recommend that patients with early stage breast cancer with overexpression of human epidermal growth factor-like receptor No 2 (*HER-2*) receive trastuzumab [7], and erlotinib is recommended for epidermal growth factor receptor (*EGFR*) mutation positive nonsquamous non-small cell lung cancer [8]. Even with FDA recommendations for use of pharmacogenomic testing for these and other drugs, insurance companies can choose whether or not to cover them [1] and for whom. While pharmacogenomic tests for cancer are available, they are underutilized in clinical care [9,10]. 

It is critical to determine factors that could be related to this underutilization. Previous studies have examined the adoption of pharmacogenomic testing by providers [11] and attitudes of providers towards testing and subsequent decision-making for patients [12,13]. Our previous study of patients’ and providers’ views on access to cancer-related pharmacogenomic tests found that the process could be time-consuming and complex [14]. Nevertheless, no studies have examined the processes for ordering and paying for pharmacogenomic tests in the context of cancer care. The objective of this study was to explore the process of ordering and paying for pharmacogenomic tests through interviews with providers, patients, and laboratories in order to identify challenges or strategies that could facilitate access.

## 2. Materials and Methods

### 2.1. Overall Design

This qualitative study was approved by the Institutional Review Board at Harvard Pilgrim Health Care (HPHC). Provider, patient, and laboratory participants were given a $25 gift card for their time and appreciation of their participation.

### 2.2. Sampling

We used insurance claims data from HPHC, a regional health plan covering about 1 million members from New England. Based on HPHC claims data from 1 May 2013 to 30 April 2014, we identified 126 providers who had prescribed any of a selected list of cancer targeted therapies that could be informed by pharmacogenomic tests in accordance with clinical guidelines (Table 1). Details of our sampling process are described in a previous study [14]. Briefly, we mailed invitation letters to 126 providers and 261 patients, asking them to call a toll-free line or email if they were interested in participating in a telephone interview. Through our provider interviews, we learned that diagnostic laboratories play a major role in the process of access and reimbursement. Representatives from key laboratories that manufacture and run pharmacogenomic testing were recruited using snowball sampling. We identified and contacted 12 diagnostic lab employees who had experience with pharmacogenomic testing, either in a commercial or academic lab setting.

### 2.3. Instrument and Data Collection

Once selected, provider and patient interviews were conducted via telephone by two of the study’s investigators, Wu and Ceccarelli. We asked questions in four main areas: (1) the process for deciding to order this particular medicine and whether any pharmacogenomic tests were ordered before starting the medication; (2) factors influencing the decision to order or not order the test and to review the clinical implementation process for the test; (3) whether the provider discussed ordering the pharmacogenomic test with the patients and to what extent the patient was involved in the decision to order the test; and (4) challenges and difficulties in obtaining the pharmacogenomic test, whether prior authorizations were requested, whether genetic testing was ever denied, and how health insurance coverage affects use of pharmacogenomic tests.

Interviews with representatives of diagnostic labs were conducted via telephone by two of the study’s investigators, Lu and Loomer. Questions focused on similar content to provider and patient interviews: (1) the process of conducting and billing for pharmacogenomic tests in the context of cancer; (2) factors that influenced communication with patients, providers, and insurers; (3) how providers or insurers were involved in the testing or billing process, at any stage; and (4) challenges in conducting and billing tests including prior authorizations and denials of coverage. The interview questions served as a guide, and the interviewer had discretion in phrasing, using probes, and posing additional questions during the interview. Interviews lasted approximately 30 to 45 min each.

### 2.4. Analysis

The initial coding scheme was developed by the study team using the interview questions as a starting point. While provider transcripts were analyzed by Wu and Ceccarelli, patient and lab transcripts were analyzed by Wu and Loomer. Themes were identified while building the codebook. Findings were circulated to the study team, discrepancies discussed until agreed upon, omissions identified, and the codebook and coding scheme revised and finalized. Transcripts were again coded using the finalized codebook and coding scheme.

## 3. Results

**Figure 1:**
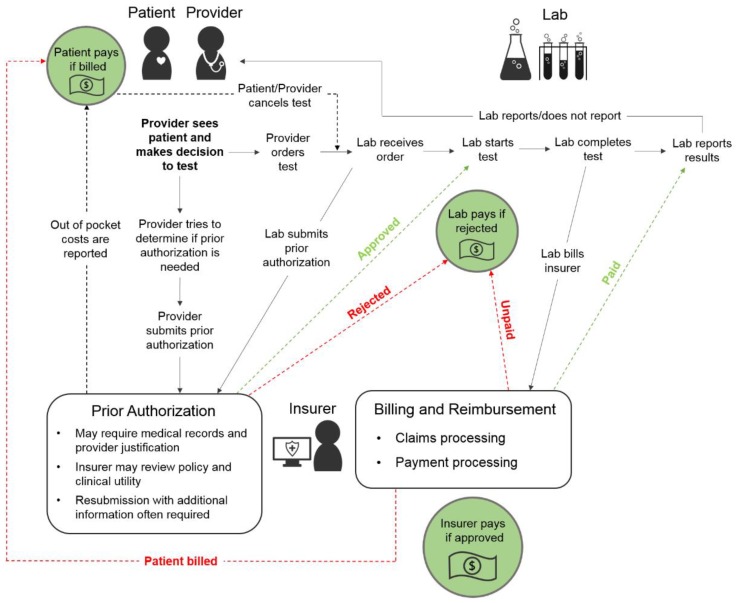
Schematic of the clinical implementation process for pharmacogenomic tests.

Table 1 provides an overview of interview participants. Based on results of the interviews of the providers, patients, and representatives of laboratories, we developed a diagram of the processes involved in ordering and paying for pharmacogenomic tests (Figure 1). After a patient receives a diagnosis, a provider orders a pharmacogenomic test. Prior to ordering the test, providers may determine if prior authorization is needed. Prior authorization may require the provider to provide justification; the insurer may review clinical utility policies to decide on approval. Once the provider orders the test, the lab receives the order. Sometimes the lab submits the prior authorization to the insurer. The lab usually starts the testing process immediately after receiving the order. The lab then completes the test and reports results to the provider. Once the lab test is completed, the lab bills the insurer. Sometimes the patient pays for the test partially or fully, sometimes the insurer covers the costs of the test fully, and sometimes the laboratory absorbs the costs of the test if the insurer does not pay and the laboratory has conducted the testing. 

### 3.1. Challenges, Adaptations, Impact, and Recommendations

Interviewees reported on challenges, adaptations, impact, and recommendations for each stage in the ordering process of pharmacogenomic testing (Table 2). The stages in the process include ordering the tests, the analysis of the samples by the laboratory, the reporting of results by the laboratory, and payment for the tests.

#### 3.1.1. Ordering Tests

Providers reported that the clinical implementation process for pharmacogenomic testing is time-consuming and variable for each insurer and patient. One provider stated, “It’s this waiting game for [the patient]”. Another challenge mentioned that it was the providers not having the expertise to order the tests, particularly panel tests. Providers noted that molecular pathology is confusing. Moreover, with so many new sequencing tests becoming available, “it is confusing to clinicians”. Some laboratories felt that larger, panel tests are sometimes ordered when they are unnecessary. A laboratory representative also stated that it is difficult to assess whether results of tests are used appropriately.

Laboratory representatives also believed that patients sometimes did not know whether the tests would be covered by insurers. Furthermore, providers stated that insurance requirements for coverage should be transparent and easy to find. Another provider expressed, “you see so many different patients with so many different insurance plans and you know different prescription plans and you don’t know what’s covered, what’s not and a large part of our day is spent you know figuring out what’s covered what’s not, what needs a prior authorization, what doesn’t, what the insurance will cover”.

One provider stated that their clinical group has adapted to the confusion around ordering and payment of pharmacogenomic tests by making resources about the process and costs available for all parties to help understand this complex field. However, laboratory representatives felt that sometimes providers order pharmacogenomic tests that are not needed.

Interviewees offered multiple suggestions for improving the clinical implementation process for pharmacogenomic tests. First, laboratory representatives felt that policies for pharmacogenomic testing could govern their use instead of insurers’ prior authorization requirements: “The benefit of developing a good medical policy that’s very clear and takes a long time is that it precludes the need for the complex and expensive preauthorization process”. Secondly, laboratory representatives stated that clinical utility needs to be established. One representative stated, “So we’re in a position now where we need to come to some form of consensus, certainly publish more to support doing panels in cancer patients and then that should be a covered service. It goes hand in hand with getting paid and having insurance pay for it, is developing clinical utility”. Third, providers felt that streamlining the process for ordering pharmacogenomic tests would be beneficial and would help prevent stress for patients as “They’re dealing with a very difficult diagnosis and it’s so stressful as it is and then to you know have patients be told well you know your insurance might not cover this or we have to find an alternative is really tough to tell people as a provider”.

#### 3.1.2. Laboratory Analysis of Samples 

Laboratory representatives stated that most laboratories start testing as soon as they receive samples in order to avoid delaying patient care. One laboratory representative stated, “within a day or so, ya’ know FedEx drops off the sample, whether that is blood and/or saliva, and the test is—we start to run the test immediately as long as it is a good sample.” Another laboratory representative stated, “We never want to put the patient in a bad place. We generally proceed with the test and end up not getting paid”. In addition, laboratory representatives noted that the process of testing is different than the process of ordering medications, although similar processes are adopted for both. For example, once laboratories start the process of pharmacogenomic testing, it is difficult to stop; thus, tests are sometimes run without knowledge of where the payment will come from, with the result that costs may end up being absorbed by the laboratory or paid for by the patient. In order to adapt, laboratories are starting to request that patients sign an agreement to cover the costs if the insurers will not. Laboratories also work hard to obtain prior authorizations and try to avoid negotiating competitive flat rates.

#### 3.1.3. Reporting of Results

Once laboratories report the results of a test, providers and patients sometimes do not know what to do with the results. Providers noted that sometimes the clinical utility of tests is not known to them, making it difficult to know how to interpret test results even if there is promise that a test result will result in improved response to treatment. One provider stated, “In today’s day and age we actually have drugs that target, that are actually useful in that situation [of a positive pharmacogenomic test] so finding that information today would actually affect how we treat a patient…. We can say that there are treatments that may improve your outcomes but are not proven to”. Laboratories have adapted by working with doctors to interpret results with one representative stating, “We are trying to sort of pull the patient and doctor through to the other side to make the results of our testing even more useful”. Laboratory representatives emphasized that providers need to understand how to interpret results with one representative stating, “They’re the ones talking to patients and if they’re telling the patients ‘this is why I’m sending the test’ and that’s not why they’re sending the test, there’s a lot of confusion on the patient’s behalf”.

Laboratories have also worked to try to inform insurers of the utility of tests. One representative stated, “We can share with payers if they’re asking about a specific gene, why we’re running that test, we have a lot of references already pulled together to share”. The reason for sharing information is “a lot of the health plans don’t have the internal expertise to know how to create an internal policy on molecular pathology”. Another laboratory representative stated they have worked to make the results of testing more useful: “And so we’ve really spent a lot of our recent efforts over the last two years or so in trying to create that bridge from the results the doctor gets to the actual treatment of the patient”. 

#### 3.1.4. Payment for Tests

Multiple providers and laboratory representatives expressed that insurance policies and requirements for prior authorization vary across insurers, and costs are mostly unknown. One laboratory representative stated, “And it’s the complexities of the insurance system that you know, we have to know every possible insurers policies or rules, for every patient in real time”. Pharmacogenomic tests can be paid for by the insurer or the patient or absorbed by the laboratories performing the testing as insurance policies vary. One laboratory representative stated, “I’ve definitely had problems with of insurance either not covering it or requiring prior authorization”. A provider stated, “I think more of an issue has been few patients who have had large bills sent to them because they were told by the insurance they can only go to certain labs to get those tests done. That’s what I’ve come across more than anything”. Another provider said, “the testing, it should be more transparent and easier for the patient and the doctors to know whether a certain test is covered by a certain insurer”. Laboratory representatives also reported that billing codes are unclear and open to different interpretations, with one representative stating, “the molecular pathology billing landscape is very bizarre…. Some people are interpreting it as, if my test has 5–50 genes, I bill the small panel code, if my test has 51 or more, I bill the big panel code. Whereas other places are interpreting it as, if I’m running a test of 300 genes, it doesn’t matter how many genes are on the test, if only four of them are clinically relevant, or if only six of them are clinically relevant. So that’s the approach that our institute has taken, only bill for the clinically relevant genes. So we run the same panel every time, it’s a panel of—I don’t even know—300, 400, 500 genes, but it’s a lot of genes”.

Providers can adapt by using vendors who charge insurers so that the hospital or providers do not receive a bill. Some laboratories have an appeals group who may reach out to the clinical team for more clinical information in order to appeal to the insurer. Another laboratory is developing a real-time adjudication process to let patients know how much their out of pocket costs would be as “the computer can generate what the patient responsibility is”.

Interviewees offered several suggestions for improving the process of payment for pharmacogenomic tests. Providers suggested making the forms easier to complete, and making it obvious which form is needed. For example, one provider stated, “You know to be honest with you in terms of prior authorization, that’s the biggest hindrance to be honest with you is that there’s no standardization of the form. So most of the time when you’re trying to obtain a prior authorization, it’s trying to figure out what form you need to submit”. Another laboratory representative stated that although universal coverage documentation would be a potential solution, “it’s just a little complicated figuring out what the right national policy would be”.

## 4. Discussion

Many pharmacogenomic tests are currently available and thousands are in the pipeline for potential future use, yet little is known about their clinical implementation process. Our study found that the clinical implementation process for pharmacogenomic tests is currently complex and variable. Providers and laboratory representatives report many challenges to the process: the lack of standardized processes makes ordering confusing, ordering is time consuming, testing is expensive, providers do not have expertise in using pharmacogenomics tests, and billing codes are nonspecific. Recommendations proposed include making the process straightforward, making insurer policies transparent, and developing clinical guidelines [15].

Some of the challenges in obtaining pharmacogenomic testing reported in our study have been seen in previous studies. For instance, Miller et al. also reported that a major challenge to ordering pharmacogenomic tests is that there is no set process for ordering the tests [16]. They also noted that many providers reported that a lack of knowledge about how to order pharmacogenomic testing for cancer was an important barrier [16]. Moreover, providers and laboratories in our study reported that guidelines are needed on appropriate and relevant testing. Furthermore, laboratories in our study felt an additional challenge is providers may not have the appropriate expertise to decide when to order tests. While other studies support some of our results that providers lack confidence in this area, our qualitative interviews allowed a broad exploration of additional challenges rather than being limited to survey questions that were asked [16,17]. Providers and laboratory representatives in our study identified additional challenges such as ordering pharmacogenomic tests is time-consuming, there is great variability in insurance coverage, testing is expensive, clinical utility of tests is difficult to assess, and ordering of panel tests is more complicated than single gene assays. Furthermore, our study also highlights the complexity that the prior authorization process adds to the process.

Our findings that some providers lack the expertise to order the tests, and that health care providers lack adequate training in genomics, especially given that staying on top of the latest available genomic medicine tools, is a challenge are also consistent with prior research [1,18,19] A study by Weldon et al. used qualitative interviews conducted from 2008 to 2009 that focused on breast cancer pharmacogenomic tests concluded that major barriers to ordering the tests were poor timing of testing relative to treatment decisions guided by the tests and obstacles related to reimbursement [20]. Even though our study was several years later when more tests were available, these same challenges remain.

Strengths of our study include that it focuses on examining the processes of ordering pharmacogenomic tests from multiple viewpoints, i.e., providers, patients, and representatives of diagnostic laboratories. Understanding such processes while there are already many cancer-related pharmacogenomic tests being used—but prior to the tremendous influx of available tests—could help guide use and payment of pharmacogenomic tests for a wide range of diseases. Despite the strengths of our study, a few limitations deserve mention. First, our study is limited to patients who are insured by Harvard Pilgrim Health Care and their providers and laboratories but we also included commercial labs that serve other insurers and providers. Secondly, all the pharmacogenomic tests were cancer-related and may not be generalizable to other pharmacogenomic tests. We focused on cancer-related tests because they are being used in current clinical care. In addition, interviewing insurers was beyond the scope of this study.

In summary, processes related to ordering and obtaining insurance coverage for pharmacogenomic tests varies greatly across institutions and is time-consuming. Potential solutions include streamlining the process for ordering tests, making reimbursement requirements by insurers transparent, developing guidelines, and improving communication between providers, patients, and laboratories.

## Figures and Tables

**Table 1:** Interview participants and demographic information.

**Stakeholder Group**		
Provider	10	
Patient	16	
Diagnostic Lab	8	
**Sex**		
Female	20	
Male	14	
**Ethnicity/Race**		
Asian	4	
White	29	
More than One Race	1	
**Age**		
30–39 years	4	
40–49 years	8	
50–59 years	14	
Over 60 years	8	
Cancer Type *	Provider	Patient
Breast	3	5
Colorectal	2	0
**Non-Small-Cell Lung Cancer**	5	3
Prostate	0	2
Leukemia	3	4
Other	4	5
**Drug^**		
Cetuximab	1	2
Panitumumab	0	0
Trastuzumab	1	5
Pertuzumab	1	0
Ado-Trastuzumab Emtansine	1	0
Lapatinib	1	0
Trametinib	2	0
Dabrafenib	2	3
Crizotinib	5	5
Dasatinib	3	2
Imatinib	2	0
Bosutinib	3	0

* Cancer diagnosis that patient received or provider diagnosed that led to inclusion in the study. ^ Cancer drug that patient received or provider diagnosed that led to inclusion in the study.

**Table 2:** Challenges, adaptations, impact, and recommendations on the pharmacogenomic testing process.

Theme *Category*	Subtheme	Quotation	Stakeholders
**Ordering** *Challenge*	Clinical implementation process is time-consuming, variable	*I have to be telling them I’m sorry. I submitted the prior authorization, I’m waiting for the insurance, I’m sure it will be approved just give me another few days and I’ll get back to you.*	Providers, Labs
**Ordering** *Challenge*	No set process for ordering; no designated staff; time consuming Providers may not have needed expertise to select the correct test	*I think the ordering physicians are sometimes confused and I think the patients are sometimes confused.*	Providers, Labs
**Ordering** *Challenge*	Multiple labs offering sequencing	*There are a lot of labs out there that now are offering something in this gene sequencing area. It seems like every day another lab pops up and one has a specific test for colon cancer and one has a specific test for breast cancer, another for pancreatic, another has this, another has that. I think it’s confusing to clinicians.*	Labs
**Ordering** *Challenge*	Patients do not know if tests will be paid for	*I think it’s confusing to patients at times because they don’t know if it’s going to be paid for.*	Providers
**Ordering** *Adaptation*	Resources made available for all parties to understand complex field	*And we have a lot of technical resources, both fields based and available on the phone, to discuss test choices with clinicians, to discuss results with clinicians, to say— if they have questions, if they get answer 1 back, if they should do another step, if I should look at something else. There are a lot of resources available. I think that that’s the way this area is built; to be very, very consultative.*	Labs
**Ordering** *Impact*	Panel tests may be unnecessarily ordered	*Ordering physicians are grabbing a lot of information and the thought is that patients will unnecessarily act upon some of this genetic information that never may manifest itself.*	Labs
**Ordering** *Recommendation*	Insurer policies need to be clinically relevant	*So recognizing that the payers couldn’t come up with it themselves, and if they came up with it themselves it probably wouldn’t be in line with what we consider to be clinically relevant.*	Labs
**Ordering** *Recommendation*	Need definitions and to establish clinical utility	Along with that comes proof of clinical utility and I think many medical directors view genetic testing currently in oncology as—outside of the danger of EGFR*, BRAF, KRAS, apart from the standard of care tests, larger panels or next gen sequencing, they don’t see the levels of evidence that they see for other covered services. And so that’s a challenge.	Labs
**Ordering** *Recommendation*	Need better information technology support	*The two big [challenges] would be IT support and informatics support.*	Labs
**Lab Analysis of Samples** *Challenge*	Unable to stop request once made	*Unfortunately, because of how molecular pathology works—and a lot of our payer’s struggle to understand this—is it’s not like giving a drug… there’s not really an easy way to stop the test [if prior authorization is not approved]. So it’s not the type of thing where we can call the payer and say this is what we’re doing, and if they say it’s not approved, we can’t really do anything about it. We can’t stop it, it’s already kind of in progress. From our perspective, it’s the first step of getting that authorization because right now we’re not getting that authorization and having to eat the cost if it comes back no prior authorization.*	Labs
**Lab Analysis of Samples** *Impact*	Lab sometimes reports test results before knowing who will pay	*Many times we will report out the test results before we can present what the patients out-of-pocket costs will be. If we determine that there are no prior authorizations, it’s a small window of time, let’s say five business days, to go back and get that because you never want the sample to go bad.*	Labs
**Lab Analysis of Samples** *Impact*	Lab does not report findings because test was not paid for	*Well once it’s [testing] done, it’s done. If it’s done in the middle, it happens very rarely I have to say— But once in a while it does and honestly, we just kind of finish it off but then we don’t report it. So that’s kind of what we do because it’s hard to stop in the middle.*	Labs
**Lab Analysis of Samples** *Adaptation*	Labs starting to ask patients to sign a waiver that they will cover the costs if the insurer does not	*we may start asking the patients to sign a waiver if we can’t get approval for it just because … there isn’t enough evidence where we would be able to confidently say we can for sure get this covered.*	Labs
**Lab Analysis of Samples** *Adaptation*	Phone calls, try to get PA	*If [prior authorization is not approved], the very next morning we make some phone calls to see, before the test-prep for the test can start but the test actually isn’t in the rigors of being done. So we can stop, make some phone calls and try to get the prior auth. or try to get the clinicians office to get the prior auth. for us.*	Labs
**Lab Analysis of Samples** *Adaptation*	Price degradation	*Absolutely, it is a competitive market and we use price degradation as more and more of these labs jump in, especially the cancer piece. If you see that price going down and down and down. … We negotiate a flat rate and that flat rate is definitely market competitive.*	Labs
**Lab Analysis of Samples** *Adaptation*	Negotiate with insurers	*We are, in some cases, trying to negotiate with payers such that we have an availability to have a little gap so that it’s not just prior authorizations. … We have been able to negotiate with some payers recently, this window [before sample goes bad] so it’s kind of prior and post-authorization.*	Labs
**Reporting of Results** *Challenge*	Clinical utility of tests difficult to assess; Difficult to determine whether results are being used appropriately	*The other aspect that makes that area more challenging is that the test itself typically isn’t the intervention that causes the benefit, it’s typically some therapy that that test informs or doesn’t inform. So if it’s a surgical procedure or a drug, it’s relatively straightforward—does that drug or procedure have the intended effect. For the diagnostic test, it’s both: does that test measure what it says it measures and then second, does measuring what it says it measures actually matter in therapy? And that gets much grittier.*	Provider
**Reporting of Results** *Challenge*	Provider may not have expertise interpreting results genetic tests	*So a better understanding on behalf of the providers so that they understand when we give them a result, that’s positive or negative, what does that really mean.*	Labs
**Reporting of Results** *Challenge*	Patients are given results without context or education	*I think that’s a very concerning area in general with patient portals because they have access to all sorts of information that they have to understand without any context or background.*	Labs
**Reporting of Results** *Adaptation*	Labs attempt to stay on top of technology	*[Our lab] is trying to, because we are connected to so many doctors, we are trying to stay up with all the technology that’s out there.*	Labs
**Reporting of Results** *Adaptation*	Labs trying to inform insurers	*We have a spreadsheet full of some of the best papers out there supporting each gene for each indication so that is something that so [some] payers… relied on our clinical experts to help create their medical policy and so we have used our approach to determine nationally, or regionally rather, what is clinically appropriate.*	Labs
**Reporting of Results** *Adaptation*	Work with doctors to interpret results and have meaningful impact on treatment decision	*And so we’ve really spent a lot of our recent efforts over the last two years or so in trying to create that bridge from results the doctors gets to the actual treatment of the patient.*	Labs
**Reporting of Results** *Recommendation*	Patients and providers given better understanding of testing/results	*It would help if more of the patients— more of the people who ordered the tests really understood what the test can and cannot tell them. So a better understanding on behalf of the providers so that they understand when we give them a result that’s positive, or negative, what does that really mean.*	
**Payment for tests** *Challenge*	Insurance policies vary, including in requirements for prior authorization; cost is unknown	*More and more plans are requiring prior authorization for genetic testing to be able to get a handle on what they’re paying for and so we see the health plans requiring PA, more and more prior authorizations, not just for cancer but for genetic testing.*	Lab
**Payment for tests** *Characteristic*	If insurer does not cover, patient covers or laboratory absorbs cost	*The biggest denials we’re seeing today are for our payers that are now requiring prior authorization. Unfortunately—well fortunately, depends on who you ask—those come back as provider liable not patient liable. So we have to just eat the cost of those. We will try to appeal them sometimes, depending upon the volume. But for the most part, there’s not much we can do about those. […] So, if the payer denies it, patient liable, we would bill the patient.*	Lab
**Payment for tests** *Challenge*	Challenge: Coding is confusing	*And then these panel codes came along and it kind of mucked up the water because panel codes are described as a next generation sequencing test of 5 to 50 genes, a next gen sequencing test of 50 or more genes which seems to imply more method than the specific gene. … So depending upon which institute you ask, people have different interpretations of that.*	Labs
**Payment for tests** *Adaptation*	Ask patients to sign waiver that they will cover costs if insurer does not	*So for commercial insurance, we will have them sign a waiver. We aren’t doing it yet today but again, everything we’re billing today, we have done extensive research to make sure that it meets a certain level of clinical evidence.*	Labs
**Payment for tests** *Adaptation*	Vendor directly charges insurance	*[When] the vendor then charges directly the insurance and bypasses the hospital getting a bill, that has been very good for pathology because they have a less of a headache of keeping track of reimbursements but it also has made it a little bit less clear for the providers when a patient runs into a problem with insurance coverage because we basically don’t get those bills within our system.*	Providers
**Payment for tests** *Adaptation*	Patients can cancel test to not incur cost	*So there is a point in time, if the results haven’t been reported, to say to the patient, this is your OOP cost. At that point, the patient will say I don’t want it. Cancel the test. And so we will cancel the test. I don’t think we give them the option. I would have to check on that.*	Labs
**Payment for tests** *Adaptation*	Compile data and discuss with insurer	*If I determine we have a payer that’s rejecting a lot of X, Y, Z test(s) and it costs me a lot to do, and they’re paying for 10% of them and we get 90% of them, then I compile that data. And then I would go back to the plan and have a conversation. And I say, let’s talk about this, let’s figure it out. Because one way or another, you have to figure out a way to get paid. Whether that’s by you or by the patient.*	Labs
**Payment for tests** *Adaptation*	Appeal	*So that appeals group will look to the literature that we’ve identified in conjunction with the clinical team. They may reach out to the doctor that specifically was treating that patient for further support of why it was clinically relevant. And then they will work with them and try and write an appeal to the insurance company.*	Labs
**Payment for tests** *Recommendation*	Concise, transparent forms	*I just wish they would make it a little easier to fill out the forms without having to go back and forth and back and forth.*	Providers
**Payment for tests** *Recommendation*	Prior authorization procedures should be easy to find	*So if all the insurance companies could get standardized in terms of the form, that would make it so much easier for the patients and for us. Some what you do is submit online, some you have to fill out a form and then you have to fax it, it’s even that varies.*	Labs
**Payment for tests** *Recommendation*	Real-time adjudication	*We’ve rolled out with some payers in some regions and with a couple of the national payers, that is called real time adjudication which would do that so it doesn’t charge the patient. It does that computer connection, it pings the payer system right from before the patient gets their blood draws … So the real time adjudication is going to be the answer because then it will be specific for me as a patient, versus you as a patient.*	Labs
**Payment for tests** *Recommendation*	Universal coverage documentation	*[Some] groups are really pushing for a national coverage document instead of these local coverage determinations. But people are tentative about that though because there is so much variation and we would want to make sure they went with one that was in agreement with our assessment versus some of them that are not in agreement with what we consider clinically relevant.*	Labs
